# Supplementation With Zinc Proteinate Increases the Growth Performance by Reducing the Incidence of Diarrhea and Improving the Immune Function of Dairy Calves During the First Month of Life

**DOI:** 10.3389/fvets.2022.911330

**Published:** 2022-07-01

**Authors:** Yeqianli Wo, Yuhang Jin, Duo Gao, Fengtao Ma, Zhu Ma, Zhuo Liu, Kangkang Chu, Peng Sun

**Affiliations:** ^1^State Key Laboratory of Animal Nutrition, Institute of Animal Science, Chinese Academy of Agricultural Sciences, Beijing, China; ^2^Beijing Dairy Cow Center, Beijing, China; ^3^Shijiazhuang Junlebao Dairy Co., Ltd., Shijiazhuang, China

**Keywords:** zinc proteinate, dairy calf, diarrhea, antioxidant status, immunity

## Abstract

Two experiments were conducted to identify the optimal dose of zinc proteinate (ZP) in the diet for dairy calves and then to compare early supplementation with the ZP or zinc methionine (ZM) on the growth performance, incidence of diarrhea, antioxidant status, and immune function of dairy calves during their first month of life. In Experiment 1, forty newborn female Holstein dairy calves were randomly divided into four groups (*n* = 10): a control group (without ZP supplementation, ZP0) or groups that received ZP supplementation at 40, 80, and 120 mg zinc/day, respectively (ZP40, ZP80, and ZP120). The experiment lasted 14 days, and the growth performance, incidence of diarrhea, and serum zinc concentration were analyzed. In Experiment 2, thirty-six newborn female Holstein dairy calves were randomly allocated to three groups (*n* = 12): a negative control group (without zinc supplementation, CON), a positive control group (supplemented with 80 mg zinc/day in the form of zinc methionine, ZM), and a ZP group (supplemented with 80 mg zinc/day in the form of ZP). The experiment lasted 28 days, and the growth performance, incidence of diarrhea, serum zinc concentration, serum antioxidant indicators, and concentrations of plasma immunoglobulins and cytokines were determined on days 7, 14, 21, and 28. Results showed that in Experiment 1, supplementation with ZP to yield 80 mg zinc/day increased the ADG (*P* < 0.01) and serum zinc concentration (*P* < 0.01), and decreased the F/G (*P* < 0.01) and the incidence of diarrhea (*P* < 0.05) during days 1–14. In Experiment 2, compared with the CON group, ZP increased the ADG (*P* < 0.01), serum zinc concentration (*P* < 0.01), and plasma immunoglobulin G (IgG; *P* < 0.01) and IgM (*P* < 0.01) concentrations, but reduced the incidence of diarrhea (*P* < 0.01), serum malondialdehyde (*P* < 0.01), and plasma interleukin-1β (*P* < 0.01) concentrations during days 1–28. Overall, ZP supplementation to yield 80 mg zinc/day improves the growth performance and immune function, and decrease the incidence of diarrhea of dairy calves, which was comparable to the same dose of zinc in the form of ZM.

## Introduction

Diarrhea during the first month of life is one of the most important factors for affecting the morbidity and mortality of dairy calves (1). During this period, the innate immunity of calves remains immature, and the efficacies of antibodies transferred from the colostrum are diminishing (2). Therefore, calves are very susceptible to pathogens in the environment, which might induce an attack of diarrhea (3). It is reported that the first 2 weeks after birth is when the prevalence of diarrhea peaks in calves (4). Importantly, calves that survive from diarrhea have been found more susceptible to other diseases especially bovine respiratory disease, and they often show growth retardation and poor performance in the first lactation (5). Therefore, it is necessary to improve the immune function for newborn calves during the early stage of life.

As an essential micronutrient, zinc is required for the optimal immunity of dairy calves (6). Almost all the immune cells including neutrophils, macrophages, natural killer cells, T cells, and B cells are zinc-dependent; hence, zinc serves as a gatekeeper for the proper activation of innate and adaptive immunity (7). Zinc deficiency in dairy calves can impair immune function and exacerbate infectious diseases such as diarrhea and malaria (8). Accumulated studies have shown that supplementation with zinc enhances the growth performance and immunity of calves (9–12). Furthermore, organic zinc is generally considered to be more bioavailable than inorganic forms, owning to its enhanced ability to promote the expression of tight junction proteins and zinc transporters in the intestine (13–15).

Zinc proteinate (ZP) is produced by chelation of zinc with amino acids or partially hydrolyzed soy protein (16). ZP reportedly improves the ADG, feed intake, activities of alkaline phosphatase and superoxide dismutase (SOD) in growing lambs, and, compared with zinc sulfate, shows an enhanced ability to increase the digestibility of crude protein, organic matter, and acid detergent fiber (17). Furthermore, other studies have demonstrated that ZP effectively promotes humoral immune responses by increasing antibody levels in cows (18). However, there is little information concerning the effects of ZP on dairy calves during early life. Therefore, the aim of this study was to identify the optimal dose of ZP supplementation for dairy calves, and to further compare the effects of supplementation with the same dose of zinc in the forms of ZP and ZM on the growth performance, incidence of diarrhea, serum zinc concentration, antioxidant status and immune function during the first 4 weeks of life.

## Materials and Methods

All animal procedures were approved by the Ethics Committee of the Chinese Academy of Agricultural Sciences (Ethical Code: IAS2020-103; Beijing, China), and calves were reared in accordance with the standards of the Chinese Academy of Agricultural Sciences Animal Care and Use Committee (Beijing, China).

### Animals, Diets, and Experimental Design

Experiment 1 was conducted between February and April 2020 at Beijing Dairy Cattle Center (Beijing, China). Forty newborn female Holstein dairy calves with similar body weight (initial BW, 41.7 ± 0.7 kg) were randomly allocated to four groups (*n* = 10 per group) by a random number generator (Microsoft Corp., Redmond, WA). The four groups were as follows: a negative control group (without ZP supplementation, ZP0) or groups that received ZP at dose of 261.44, 522.88, and 784.31 mg/day, equivalent to 40, 80, and 120 mg zinc/day, respectively (ZP40, ZP80, and ZP120). ZP (Q_f_ = 357) was donated by the Mineral Nutrition Research Division, Institute of Animal Science, Chinese Academy of Agricultural Sciences (Beijing, China). The experiment lasted 14 days. The pelleted starter diet (Beijing Sino Livestock Development Co., Ltd.) was supplied to the calves during days 7–14 of the experiment. The nutritional composition of the starter is shown in Table 1.

**Table 1:** Nutrient compositions of the milk and starter diet (%, unless noted)^1^.

**Item**	**Content (%)**
**Experiment 1**	
**Milk composition (dry matter basis)**	
Density, kg/L	1.03
Protein	3.25
Fat	4.02
Lactose	4.91
Total solids	12.56
Zinc, mg/kg	3.35
**Nutrient composition of starter (dry matter basis)**	
Dry matter	89.04
Crude protein	24.45
Ether extract	1.68
Acid detergent fiber	6.85
Neutral detergent fiber	12.20
Zinc, mg/kg	175.00
**Experiment 2**	
**Milk composition (dry matter basis)**	
Density, kg/L	1.03
Protein	3.20
Fat	4.16
Lactose	5.10
Total solids	12.97
Zinc, mg/kg	3.35
**Nutrient composition of starter (dry matter basis)**	
Dry matter	91.11
Crude protein	20.70
Ether extract	4.14
Acid detergent fiber	11.31
Neutral detergent fiber	18.72
Zinc, mg/kg	136.00

1 *Analyzed value*.

Experiment 2 was conducted between September and October 2020 at Hebei Junxing Farm (Xingtai, Hebei Province, China). Thirty-six newborn female Holstein dairy calves with similar body weight (initial BW, 39.5 ± 0.7 kg) were randomly allocated to three groups (*n* = 12 per group). The three groups were as follows: (1) negative control group, given raw milk and starter only (CON); (2) ZM group, given an additional 467.88 mg ZM/day, equivalent to 80 mg zinc/day, and (3) ZP group, given an additional 522.88 mg ZP/day, equivalent to 80 mg zinc/day. ZM (purity ≥95.00%) was obtained from an independent distributor. The dose of ZP was provided based on Experiment 1, and dose of ZM was based on previous studies (9, 10, 12). The experiment lasted 28 days. The pelleted starter provided by ABNA Feed (Shanghai) Co., Ltd., was supplied to the calves during days 7–28, and the nutritional composition of the starter is shown in Table 1.

Each dam in parturition was monitored by a specialized person in Experiment 1 and 2. Calves were removed from dams immediately after birth and housed in individual pens (Experiment 1: 2.0 × 1.5 m; Experiment 2: 1.8 × 1.4 m). All calves received 4 L colostrum from a bottle within 1 h of birth. The concentration of IgG in the colostrum was tested by an optical refractometer (HT113ATC; Httechltd Tianyuan Optical Instruments Co., Ltd., China). If the concentration of IgG ≥50 mg/ml, the colostrum would be fed to newborn calves. After 1 day of age, the calves were supplied 4 L raw milk twice daily (at 08:30 and 16:00 h; total of 8 L/day). The nutritional compositions of the milk in Experiment 1 and 2 are shown in Table 1. All animals had free access to fresh water and starter concentration. If the residual starter was found <20 g on morning feeding, an additional 100 g of starter was supplied the next day to ensure adequate starter for dairy calves.

During the experimental period, organic zinc was provided once a day at 08:30 h. The appropriate dose of ZP or ZM was dissolved in 200 ml colostrum or milk in the bottle, and then fed to the calves during the morning feeding. Afterwards, more colostrum or milk was offered to the calves.

### Sampling and Analysis

The samples of milk in the two experiments were collected once per week. A 50-ml aliquot of each milk sample was mixed with a preservative (Bronopol Tablet, D&F Control System, San Ranmon Inc., Dublin, ON, Canada) before storing at −20°C. The milk composition (density, protein, fat, lactose and total solids) was analyzed by infrared analysis (Foss MilkoScan 2000, Foss Food Technology Corp., Eden Prairie, MN, USA).

Samples of starter were collected once per week during the study period. All samples were dried at 65°C for 48 h, ground by a fodder grinder, and passed through a 1-mm screen. Dry matter (DM) content was determined by oven drying to a constant weight (105°C, 4 h). Then standard procedures of the Association of Official Analytical Chemists were utilized for the determination of DM (19), and crude protein (20) content. The NDF and ADF contents were analyzed by the methods described by Van Soest et al. (21). The zinc concentration in the milk and starter were determined by inductively-coupled plasma optical emission spectroscopy (ICP-OES), as described in the Chinese National Standards (GB 5009.268, China, 2016) with some modifications (14).

The body weight of all the calves was measured after birth and before morning feeding at weekly intervals (Experiment 1: on day 7 and 14; Experiment 2: on day 7, 14, 21, and 28) using a digital scale (Tru-Test, Mineral Wells, TX). The wither height, body length and heart girth were measured after birth and before morning feeding (08:30 h) at weekly intervals. The intake of milk and starter was recorded every day, and total feed intake (sum of the DM of milk and starter intake) was measured to calculate the average daily feed intake (ADFI, dry matter basis) and feed to gain ratio (F/G) during the experimental period. The total zinc intake was also calculated by summing the intake of zinc from milk, starter and zinc supplementation.

Fecal consistency, which was recorded daily by a specialized person who was blinded to the treatments, was scored according to a 4-point scale, as reported previously (22). Briefly, 1 point represented normal consistency, 2 points represented pasty, 3 points represented semi-liquid, and 4 points represented liquid with abnormal colors. A fecal score of 1 or 2 was considered healthy, but a fecal score of 3–4 for 2 successive days was defined as diarrhea. The incidence of diarrhea was calculated using the following formula: incidence of diarrhea (%) = number of calves with diarrhea × number of days with diarrhea / (number of calves in each group × length of the trial in days) × 100%.

Blood samples were obtained from each calf *via* jugular vein puncture and collected in vacutainer tubes with or without heparin sodium (BD Biosciences, San Jose, CA) before morning feeding (Experiment 1: on day 14; Experiment 2: on day 7, 14, 21, and 28). The blood samples were centrifuged for 15 min at 3 000 × *g* and 4°C using a high-speed freezing centrifuge (Eppendorf 5810R; Eppendorf AG, Hamburg, Germany). The serum and plasma were collected and stored at −80°C. The concentration of serum zinc of each calf was determined over the experiment (Experiment 1: on day 14; Experiment 2: on day 7, 14, 21, and 28) by ICP-OES. In Experiment 2, the activity of serum SOD (A001-1), the concentration of malondialdehyde (MDA; A003-1), metallothionein (MT; H132) and the total antioxidant capacity (T-AOC; A015-2-1) were determined weekly (on day 7, 14, 21, and 28) using commercial assay kits (Nanjing Jian Cheng Bioengineering Institute, Nanjing, China). All procedures were performed according to the manufacturer's instructions. The concentrations of plasma immunoglobulin G (IgG; E11-118), IgA (E11-131), and IgM (E11-101) were determined weekly by bovine ELISA kits provided by Bethyl Laboratories (Montgomery, TX), according to the manufacturer's instructions. The concentrations of plasma cytokines including interleukinin-1β (IL-1β; JYM0006Bo), interleukinin-6 (IL-6; JYM0007Bo), interleukinin-8 (IL-8; JYM0035Bo), transforming growth factor-β (TGF-β; JYM0034Bo), and interferon-γ (IFN-γ; JYM0002Bo) were analyzed weekly with bovine ELISA kits provided by Wuhan Colorful Gene Biological Technology Co., Ltd. (Wuhan, China), following the manufacturer's instructions. The Sandwich-ELISA was used, and the absorbance at 450 nm was measured spectrophotometrically. The data was expressed as optical density (OD) units.

### Statistical Analysis

All the data were performed using SAS (version 9.4; SAS Institute Inc., Cary, NC) with data checked for normality first. The randomness of the initial BW was confirmed using the Durbin-Watson test. The incidence of diarrhea was analyzed by logistic regression (using the GENMOD procedure) and a binomial error distribution with diet as a fixed effect. The link function was a logit transformation. The result of logistic regression was then converted back to original natural units. The statistical model for final BW, withers height, body length, and heart girth included treatment as fixed effects, with initial BW, withers, body length, and heart girth as covariate. Linear and quadratic polynomial contrasts were analyzed using the CONTRAST statement of SAS. The data of growth performance (ADG, ADFI, F/G, starter intake, and zinc intake), SOD, MDA, MT, T-AOC, IgA, IgG, IgM, IL-1β, IL-6, IL-8, TGF-β, and IFN-γ were analyzed by MIXED procedure, with calf as random effect, treatment, day, and interaction between treatment and day as fixed effects, and day as repeated effect. The data were illustrated as the least square mean and standard error of the mean. Differences with *P* < 0.05 were considered statistically significant.

## Results

### Growth Performance and Incidence of Diarrhea

In Experiment 1, the effects of different doses of ZP on the body size measurement, growth performance, and incidence of diarrhea in dairy calves are summarized in Table 2. There were no differences in initial BW and body size measurement in the four groups (*P* > 0.05). Supplementation with ZP to yield 80 and 120 mg zinc/day significantly increased the final BW (*P* < 0.01) and ADG (*P* < 0.01) compared with the ZP0 and ZP40 groups. Furthermore, the ADG was linearly increased (*P* < 0.01) and the F/G was linearly decreased (*P* < 0.01), although no differences were observed in average daily feed intake by ZP supplementation. As expected, supplementation with ZP linearly increased the total zinc intake of dairy calves with the increasing dose of ZP (*P* < 0.01). But there were no differences in starter intake (*P* > 0.05) and starter zinc intake (*P* > 0.05) among groups.

**Table 2:** Effects of zinc proteinate (ZP) on the body size measurement, growth performance and incidence of diarrhea in dairy calves during the first 2 weeks of life (Experiment 1).

**Item**	**Treatment** ^**1**^				**SEM**	***P-*** **Value**		
	**ZP0**	**ZP40**	**ZP80**	**ZP120**		**Linear**	**Quadratic**	**Treatment**
	**(*n* = 10)**	**(*n* = 10)**	**(*n* = 10)**	**(*n* = 10)**				
Initial BW, kg	39.9	43.3	42.4	41.0	0.72	–	–	0.36
Final BW, kg	48.9^b^	49.1^b^	50.1^a^	50.1^a^	0.29	–	–	<0.01
Initial withers height, cm	78.3	80.5	79.4	80.1	0.53	–	–	0.50
Initial body length, cm	72.8	72.4	73.1	72.1	0.58	–	–	0.94
Initial heart girth, cm	77.0	78.7	79.1	79.0	0.39	–	–	0.17
Final withers height, cm	80.6	82.3	82.0	82.0	0.77	–	–	0.14
Final body length, cm	74.7	73.8	75.6	76.1	1.32	–	–	0.32
Final heart girth, cm	78.9	81.0	81.5	81.9	0.86	–	–	0.10
Average daily gain, g/Day	422.57^b^	437.15^b^	504.57^a^	512.46^a^	14.60	<0.01	0.82	<0.01
Average daily feed intake, g DM/Day	969.07	967.57	984.48	980.73	6.89	0.10	0.87	0.23
F/G	2.31^b^	2.23^b^	1.96^a^	1.94^a^	0.06	<0.01	0.72	<0.01
Starter intake, g DM/Day	23.66	22.08	30.50	29.21	3.81	0.15	0.97	0.33
Starter zinc intake, mg DM/Day	4.14	3.89	5.34	5.11	0.67	0.15	0.97	0.33
Total zinc intake, mg/Day	7.31^d^	47.03^c^	88.53^b^	128.30^a^	0.68	<0.01	0.98	<0.01
Incidence of diarrhea, %	27.38^a^	22.62^a^	16.67^b^	14.88^b^	0.17	0.01	0.63	0.02

a−d *Mean values in the same row with different superscripts are significantly different (P < 0.05)*.

1 *Treatment: ZP0, ZP40, ZP80 and ZP120: 0, 40, 80, and 120 mg zinc/day of zinc proteinate were supplemented, respectively*.

In Experiment 2, the effects of supplementation with ZP and ZM to yield 80 mg zinc/day on the body size measurement, growth performance, and incidence of diarrhea in dairy calves are summarized in Table 3. No variations were observed in initial BW and body size measurement between three groups (*P* > 0.05). However, supplementation with ZP and ZM significantly increased the ADG (*P* < 0.01), resulting in a higher final BW (*P* < 0.01) of dairy calves compared with CON. Furthermore, the total zinc intake was also higher in organic zinc groups than the CON group (*P* < 0.01).

**Table 3:** Effects of organic zinc sources on the body size measurement, growth performance and incidence of diarrhea in dairy calves during the first month of life (Experiment 2).

**Item**	**Treatment** ^**1**^			**SEM**	***P-*** **Value**		
	**CON** **(*n* = 12)**	**ZP** **(*n* = 12)**	**ZM** **(*n* = 12)**		**Treatment**	**Days**	**Treatment × days**
Initial BW, kg	39.55	40.55	38.36	0.72	0.48	–	–
Final BW, kg	54.37^b^	58.46^a^	58.67^a^	1.23	<0.01	–	–
Initial withers height, cm	74.33	77.00	74.42	0.56	0.09	–	–
Initial body length, cm	64.92	66.33	65.08	0.52	0.50	–	–
Initial heart girth, cm	78.83	81.00	78.83	0.49	0.11	–	–
Final withers height, cm	83.92	86.00	86.66	1.45	0.13	–	–
Final body length, cm	76.20	78.31	77.41	1.40	0.33	–	–
Final heart girth, cm	92.06	95.71	95.56	1.00	0.10	–	–
Average daily gain, g/Day	531.36^b^	672.40^a^	690.91^a^	28.28	<0.01	0.45	0.14
Average daily feed intake, g DM/Day	910.11	969.19	988.32	21.37	0.21	0.49	0.05
F/G	1.85	1.70	1.46	0.14	0.16	0.13	0.38
Starter intake, mg DM/Day	41.51	47.23	48.22	3.80	0.41	<0.01	0.42
Starter zinc intake, g DM/Day	7.27	8.27	8.43	0.67	0.41	<0.01	0.42
Total zinc intake, mg/Day	10.17^b^	91.60^a^	91.36^a^	0.66	<0.01	<0.01	0.29
Incidence of diarrhea, %	26.49^a^	14.58^b^	13.99^b^	0.12	<0.01	–	–

a, b *Mean values in the same row with different superscripts are significantly different (P < 0.05)*.

1 *Treatment: CON, no organic zinc supplementation; ZP group, provided 522.88 mg zinc proteinate (equivalent to 80 mg zinc/day); ZM group, provided 467.88 mg ZM per day (equivalent to 80 mg zinc/day)*.

The incidence of diarrhea of dairy calves was significantly reduced by zinc supplementation. In Experiment 1, the incidence of diarrhea was significantly lower in the ZP80 and ZP120 groups than in the ZP0 and ZP40 groups (*P* < 0.05). In Experiment 2, supplementation with ZP and ZM significantly reduced the incidence of diarrhea during the first month of life compared with the CON group (*P* < 0.01).

### Serum Zinc Concentrations

**Figure 1:**
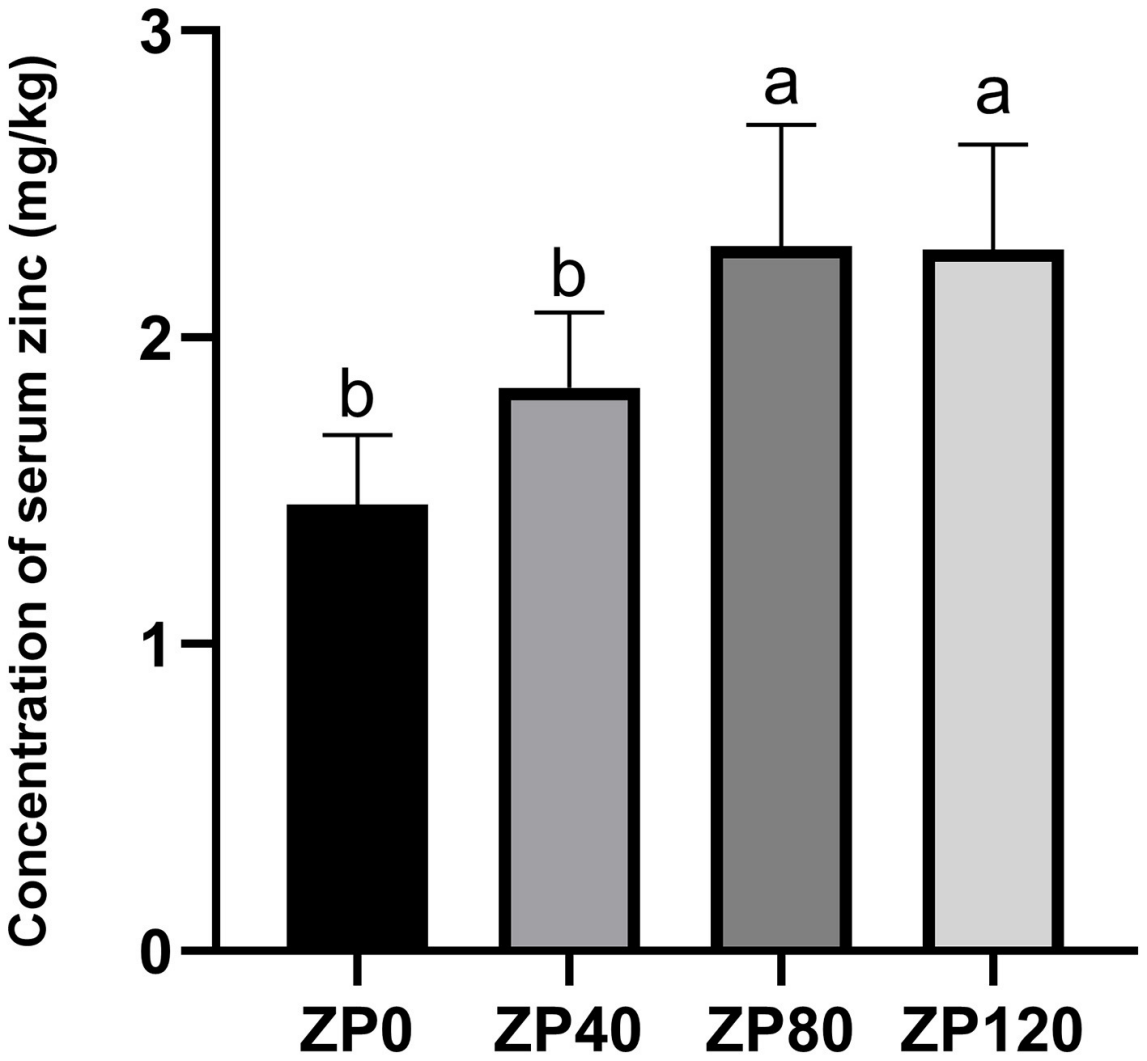
The concentration of serum zinc in dairy cows supplemented with different dose of zinc proteinate on day 14 (Experiment 1). ^a, b^Mean values in the same row with different superscripts are significantly different (*P* < 0.01). ZP0, ZP40, ZP80, and ZP120: 0, 40, 80, and 120 mg zinc/day of zinc proteinate were supplemented, respectively (*n* = 12 per group).

**Figure 2:**
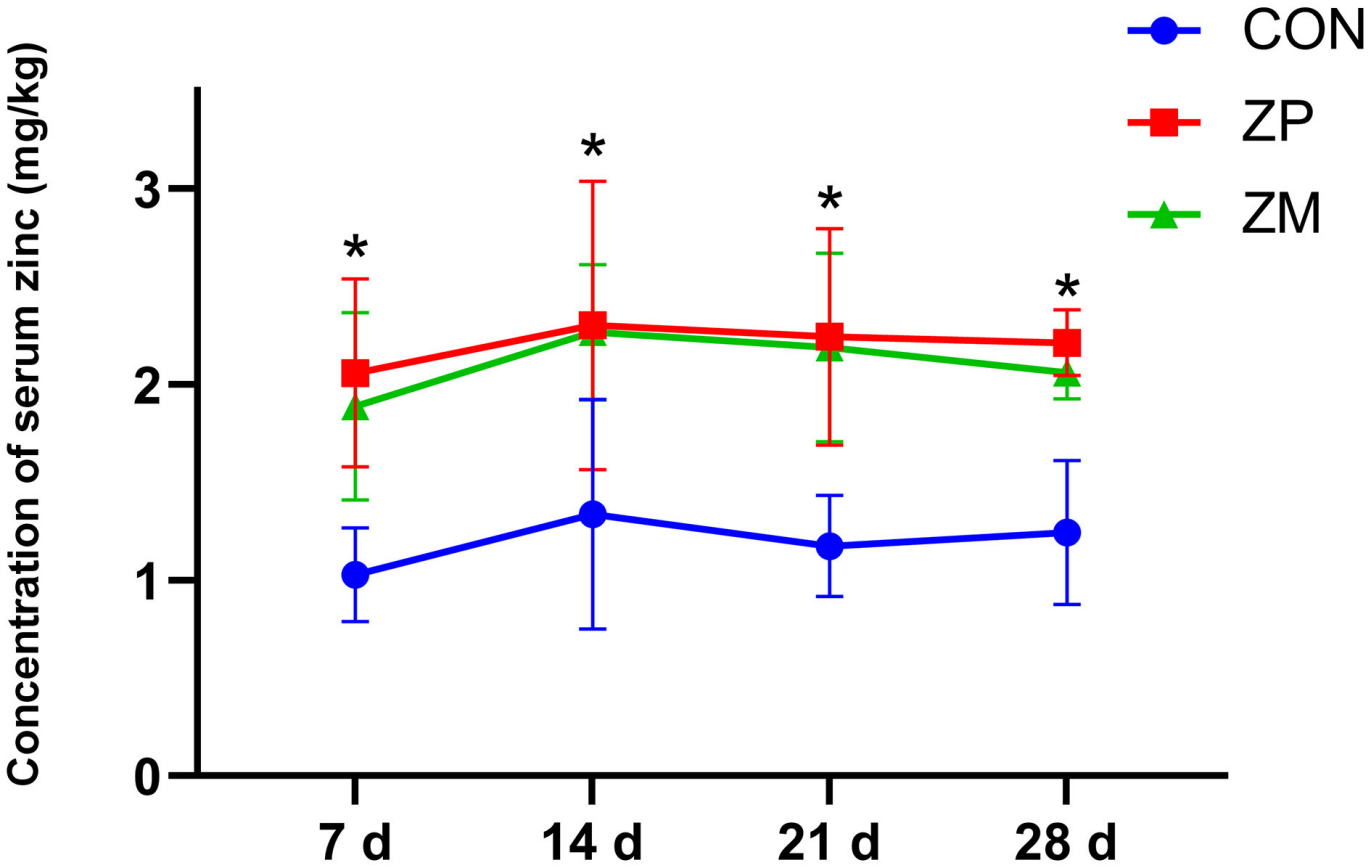
The concentration of serum zinc in dairy cows supplemented with organic zinc sources during the first month of life (Experiment 2). *Mean values in the same row with different superscripts are significantly different (*P* < 0.01). CON, no organic zinc supplementation (*n* = 12); ZP group, provided 522.88 mg zinc proteinate (equivalent to 80 mg zinc/day; *n* = 12); ZM group, provided 467.88 mg ZM per day (equivalent to 80 mg zinc/day; *n* = 12).

As shown in Figure 1, the serum zinc concentration in dairy calves was linearly increased as the supplementation doses were increased. Compared with ZP0 and ZP40 groups, the serum zinc concentrations in dairy calves from ZP80 and ZP120 were significantly greater (*P* < 0.01). Figure 2 presents the fluctuation of serum zinc concentrations after supplemented with organic forms of zinc in dairy calves during the first month of life. The serum zinc concentrations in the ZP and ZM groups were significantly higher than that in the CON group (*P* < 0.01).

### Antioxidant Property

As shown in Table 4, the serum MDA concentration was significantly lower in the ZP and ZM groups than in the CON group (*P* < 0.05). Notably, the concentrations of serum MT (*P* < 0.05) and T-AOC (*P* < 0.05) in the calves in ZM group were higher than that in the CON and ZP groups. No significant differences were observed in the serum SOD activity in the calves among the three groups (*P* > 0.05).

**Table 4:** Effects of organic zinc sources on serum antioxidant status of dairy calves during the first month of life (Experiment 2).

**Item**	**Treatment** ^**1**^			**SEM**	***P-*** **Value**		
	**CON** **(*n* = 12)**	**ZP** **(*n* = 12)**	**ZM** **(*n* = 12)**		**Treatment**	**Days**	**Treatment × Days**
Malondialdehyde, nmol/ml	3.90^a^	2.72^b^	3.07^b^	0.24	0.01	0.54	0.03
Superoxide dismutase, U/ml	72.83	74.74	75.63	0.94	0.13	0.27	0.03
Metallothionein, pg/ml	1,289.05^b^	1,359.62^b^	1,412.55^a^	26.26	0.02	0.18	0.17
Total antioxidant capacity, mmol/L	0.29^b^	0.31^b^	0.38^a^	0.02	0.03	0.29	0.43

a, b *Mean values in the same row with different superscripts are significantly different (P < 0.05)*.

1 *Treatment: CON, no organic zinc supplementation; ZP group, provided 522.88 mg zinc proteinate (equivalent to 80 mg Zn/day); ZM group, provided 467.88 mg ZM per day (equivalent to 80 mg Zn/day)*.

### Plasma Immunoglobulin and Cytokine Concentrations

As shown in Table 5, compared with the CON group, the concentration of plasma IgG was increased significantly by the administration of ZP and ZM (*P* < 0.01). Furthermore, supplementation with ZP and ZM significantly increased the concentration of plasma IgM, with that in the ZM group was significantly higher than in the ZP group (*P* < 0.01). The concentration of plasma IgA was not affected by supplementation with ZP or ZM in the present study. In addition, the concentrations of plasma IgG, IgM, and IgA were affected by the effect of day (*P* < 0.05), but no significant difference were observed in treatment × day interactions.

**Table 5:** Effects of organic zinc sources on concentrations of plasma immunoglobulin in dairy calves during the first month of life (Experiment 2).

**Item**	**Treatment** ^**1**^			**SEM**	***P-*** **Value**		
	**CON** **(*n* = 12)**	**ZP** **(*n* = 12)**	**ZM** **(*n* = 12)**		**Treatment**	**Days**	**Treatment × Days**
Immunoglobulin G, mg/ml	9.47^b^	13.02^a^	12.40^a^	0.62	<0.01	0.01	0.13
Immunoglobulin M, mg/ml	4.25^c^	5.17^b^	6.49^a^	0.26	<0.01	<0.01	0.50
Immunoglobulin A, μg/ml	136.37	151.27	168.90	11.06	0.14	<0.01	0.86

a−c *Mean values in the same row with different superscripts are significantly different (P < 0.05)*.

1 *Treatment: CON, no organic zinc supplementation; ZP group, provided 522.88 mg zinc proteinate (equivalent to 80 mg Zn/day); ZM group, provided 467.88 mg ZM per day (equivalent to 80 mg Zn/day)*.

As shown in Table 6, supplementation with ZP and ZM decreased the concentration of plasma IL-1β compared to the CON group (*P* < 0.01). The effect of day (*P* < 0.01) and treatment × day interaction (*P* < 0.01) was also observed in IL-1β. In addition, there was a treatment × day interaction in plasma IL-6 concentration (*P* < 0.05). The effects of treatment did not affect the concentrations of other cytokines including IL-8, TGF-β and IFN.

**Table 6:** Effects of organic zinc sources on concentrations of plasma cytokine in dairy calves during the first month of life (Experiment 2).

**Item**	**Treatment** ^**1**^			**SEM**	***P-*** **Value**		
	**CON** **(*n* = 12)**	**ZP** **(*n* = 12)**	**ZM** **(*n* = 12)**		**Treatment**	**Day**	**Treatment × Day**
Interleukinin-1β (pg/ml)	172.79^a^	140.42^b^	141.39^b^	4.32	<0.01	<0.01	<0.01
Interleukinin-6 (pg/ml)	83.52	84.21	85.90	1.73	0.61	0.79	0.02
Interleukinin-8 (pg/ml)	138.06	113.32	118.52	13.31	0.41	<0.01	0.99
Transforming growth factor-β (pg/ml)	378.16	372.97	387.85	19.26	0.85	<0.01	0.44
Interferon-γ (pg/ml)	71.05	81.47	81.40	3.81	0.10	<0.01	0.50

a, b *Mean values in the same row with different superscripts are significantly different (P < 0.05)*.

1 *Treatment: CON, no organic zinc supplementation; ZP group, provided 522.88 mg zinc proteinate (equivalent to 80 mg Zn/day); ZM group, provided 467.88 mg ZM per day (equivalent to 80 mg Zn/day)*.

## Discussion

Our research team is dedicated to identifying the optimal low doses of different sources of zinc to alleviate diarrhea and promote growth in dairy calves during their early life. In our previous studies, we found that supplementation with 80 mg zinc/day in the form of ZM or zinc oxide, effectively reduced diarrhea and promoted the growth of dairy calves in the first 2 weeks after birth (12, 15). Here in Experiment 1, supplementation with ZP to yield 80 and 120 mg zinc/day significantly reduced the incidence of diarrhea but increased growth performance by increasing ADG and final body weight and decreasing feed-to-gain ratio during days 1–14. Furthermore, the concentration of serum zinc increased with increasing amounts of ZP supplementation, and calves in ZP80 and ZP120 groups had higher zinc concentration than did calves in the CON and ZP 40 groups. Therefore, we identified the optimal dose of ZP supplemented in the diet of diary calves was also to yield 80 mg zinc/day, similar to our previous results (23).

As mentioned above, the first month of life is a critical period of development for dairy calves, when milk and limited starter intake might not fulfill the zinc requirements of dairy calves during the first 28 days of life (24, 25). Therefore, we investigated the potential beneficial effects of a 4-week supplementation of newborn dairy calves with ZP, compared with ZM. As expected, ZP and ZM improved the growth performance of dairy calves by increasing the ADG during the experimental period in Experiment 2. Furthermore, the incidence of diarrhea in dairy calves was also reduced by ZP and ZM supplementation, resulting in the higher final body weight of dairy calves in ZP and ZM groups compared to the CON group.

To further investigate the absorption of different organic zinc sources, the concentration of serum of zinc on days 7, 14, 21, and 28 was determined. Organic zinc sources have high bioavailability because they increased zinc absorption by the body (13, 26). Wang et al. (18) showed that amino acid chelated zinc and ZP increased the plasma zinc concentration of lactating cows. In line with the previous studies, in Experiment 2, the concentrations of serum zinc in dairy calves were increased dramatically following ZP or ZM supplementation on days 7, 14, 21, and 28, and no differences were observed between the two organic zinc sources.

Oxidative stress happens when the excessive production of reactive oxygen species overwhelms the capacity of antioxidant systems in the body (27, 28). Previous studies have shown that zinc has an antioxidant function (29), and supplementation with ZM reportedly decreases the concentration of MDA, but increases that of MT and T-AOC in the serum of ruminants (14, 17, 23), and similar results were also observed in the present study. Different from ZM, ZP did not present the same antioxidant effects as ZM because it did not affect the serum MT or T-AOC concentrations compared with the CON group. However, ZP did exhibit antioxidative property by decreasing the concentration of serum MDA in dairy calves during the first month of life. Similar results were also observed by Jin et al. (30), who indicated that ZP decreased the serum MDA of dairy calves on day 14.

The most direct connection between zinc and the immune function is its role in cell replication and proliferation, which is of great importance for maintaining the normal activity and integrity of immune cells and systems (18, 29). The IgG, IgM, and IgA are involved in defense against invading pathogens. These antibodies recognize and bind to antigens specifically, thereby activating several immune responses (3) that selectively eliminate microorganisms, viruses, and other foreign molecules (31). Zinc deficiency impairs B lymphocyte development and the production of antibodies, especially IgG (32). ZM supplementation reportedly enhanced the resistance to infection by increasing the total immunoglobulin concentration in weaning calves (29). In addition, a recent study demonstrated that ZM supplementation increased the concentrations of IgA and IgM in the colostrum of dairy cows (33). In the present study, we found that not only ZM, but also ZP increased the plasma IgG concentrations. Simultaneously, the concentration of IgM was increased in the calves in the ZM and ZP groups, with that in the ZM group higher than in the ZP group. As expected, resistance to disease and innate immunity was intensified by upregulated levels of immunoglobulins in calves after organic zinc supplementation.

As a common pro-inflammatory cytokine, IL-1β is secreted by most innate immunity cells, including neutrophils, monocytes, and macrophages. Once innate immunity cells recognize the pathogens, they would secrete IL-1β to promote inflammatory responses, including the generation of ROS to kill pathogens (34). As a result, the concentration of IL-1β could indicate the body infection. Researches have shown that zinc suppresses pro-inflammatory cytokines production, whereas zinc deficiency induces the production of pro-inflammatory cytokines such as IL-1β and IL-6 (35). Chen et al. (33) suggested that the blood concentration of IL-1 in dairy cows decreased linearly, with the dose of ZM increasing. So far, there are few studies on the effect of ZP on plasma cytokines. In this study, we found that ZP decreased the concentration of plasma IL-1β in calves, and its effect was similar to that of ZM. The results showed that the calves had low levels of inflammation and infection, and it was related to the anti-bacterial function of zinc, which inhibits lipopolysaccharide induced activation of *NF-*κ*B p65* and the production of IL-1β (36). TGF-β is an important signaling molecule in the *TGF-*β*/SMAD* signaling pathway, which plays an important role in regulating cell apoptosis, differentiation, immune function and inflammatory response (37). IFN-γ is mainly secreted by NK cells and plays a role in the innate immune process against pathogenic infection (38). However, the results of this experiment showed that there were no differences in plasma IL-6, IL-8, TGF-β, and IFN-γ concentrations between different groups, indicating that the alleviation of inflammatory by zinc was irrelevant with such signal pathways. An over production of pro-inflammatory cytokines is responsible for diarrhea induced by intestinal inflammation (39). In this study, the lower concentration of plasma IL-1β in organic zinc groups shows a similar trend to the decreasing incidence of diarrhea, indicating the better health condition of dairy calves with ZP and ZM supplementation.

## Conclusion

The present study indicated that a zinc supplementation amount of 80 mg/day in the form of ZP was the optimal dose for dairy calves during the first 2 weeks of life. The appropriate dose of ZP reduces the incidence of diarrhea, increases the growth performance and serum zinc concentration, and improves the antioxidant and immune functions in dairy calves during the first 4 weeks of life, which has similar growth-promoting and anti-diarrheal properties as ZM. This study suggests that ZP might become a prospective organic zinc source which may be recommended for dairy calves during their early stage of life.

## Data Availability Statement

The original contributions presented in the study are included in the article/supplementary material, further inquiries can be directed to the corresponding author/s.

## Ethics Statement

The animal study was reviewed and approved by Ethics Committee of the Chinese Academy of Agricultural Sciences (Beijing, China). Written informed consent was obtained from the owners for the participation of their animals in this study.

## Author Contributions

YW conducted the experiment, analyzed data, and wrote the draft of the manuscript. YJ, DG, FM, ZM, KC, and ZL conducted the experiment. PS conceptualized the experiment, supervised the project, and edited the final version of the manuscript. All authors contributed to the article and approved the submitted version.

## Funding

This study was supported by the Agricultural Science and Technology Innovation Program (cxgc-ias-07) and the National Program for Support of Top-notch Young Professionals.

## Conflict of Interest

ZL was employed by Shijiazhuang Junlebao Dairy Co., Ltd. The remaining authors declare that the research was conducted in the absence of any commercial or financial relationships that could be construed as a potential conflict of interest.

## Publisher's Note

All claims expressed in this article are solely those of the authors and do not necessarily represent those of their affiliated organizations, or those of the publisher, the editors and the reviewers. Any product that may be evaluated in this article, or claim that may be made by its manufacturer, is not guaranteed or endorsed by the publisher.
